# Cardiac Arrest Triggered by Ruptured Aberrant Left Hepatic Artery Aneurysm and Subsequent Management

**DOI:** 10.7759/cureus.81988

**Published:** 2025-04-10

**Authors:** Elizabeth Tan, Benjamin Thomson

**Affiliations:** 1 General and Breast Surgery, Northern Hospital, Melbourne, AUS; 2 Hepatobiliary and Trauma Surgery, Royal Melbourne Hospital, Melbourne, AUS

**Keywords:** aberrant hepatic artery, hepatic artery aneurysm, left hepatic artery aneurysm, ruptured hepatic artery aneurysm, splanchnic artery aneurysm

## Abstract

Hepatic artery aneurysms (HAAs) are a rare type of splanchnic artery aneurysm. Our understanding is limited given the rarity of incidence. Rupture on presentation has a high mortality rate. We report the case of a 71-year-old woman who presented with a ruptured aberrant left HAA and subsequent cardiac arrest. She had risk factors of hypertension and was a long-term smoker. Post imaging-guided diagnosis and emergent multi-disciplinary discussion with the interventional radiology team, she underwent surgical resection of the aneurysm. Her recovery was unremarkable with gradual resolution of hepatic dysfunction and no residual post-arrest deficits. This case provides insight into the challenges of HAA anatomy and decision-making regarding surgical versus endovascular management and techniques.

## Introduction

Hepatic artery aneurysms (HAAs) are the second most common type of splanchnic artery aneurysms at 20% [1,2]. With an incidence of 0.02%-0.4% [1,2], they often occur in the sixth decade of life with a male predominance [2-4]. Extra-hepatic HAA are most common (80%) with the majority in the common hepatic artery (60%) [4,5]. Risk factors include atherosclerosis, vasculitis, hypertension, smoking, connective tissue disease, and iatrogenic injury [2,3,6-8].

With a rise in advanced imaging, incidental HAAs are increasingly diagnosed [1,6,8]. However, presentations are varied with abdominal pain, a mass, upper gastrointestinal bleed [8], obstructive jaundice, and rupture [3,4]. A minority present with Quinke’s triad- abdominal pain, jaundice, and haemobilia [1].

Treatment options include open ligation versus endovascular repair [2,6-9]. In this case report, we describe the former approach with a 2.5 cm-sized HAA in a post-arrest 71-year-old woman. Further insight into the challenges of decision-making regarding management and operative approach will be discussed.

## Case presentation

**Figure 1:**
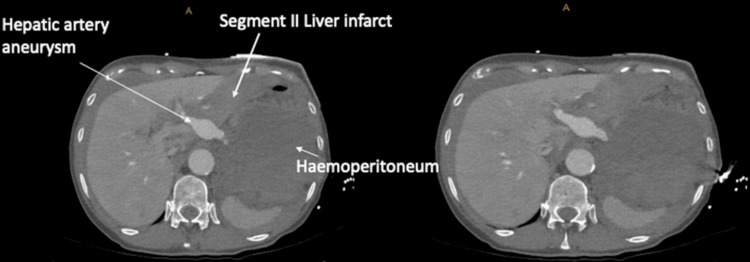
Axial phase of CT aortogram demonstrating ruptured left hepatic artery aneurysm with haemoperitoneum and segment II liver infarct.

**Figure 2:**
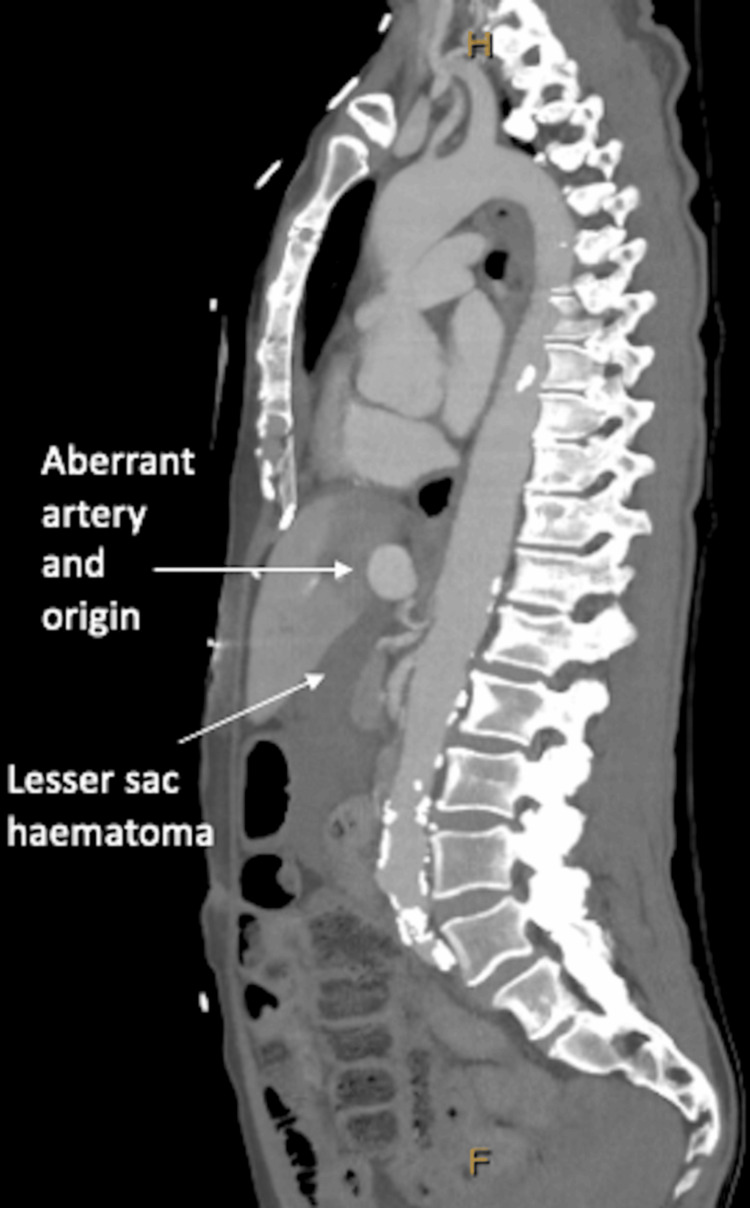
Sagittal phase of CT aortogram demonstrating aberrant left hepatic artery aneurysm and origin from left gastric artery, adjacent to coeliac axis. There is a large haematoma in the lesser sac.

**Figure 3:**
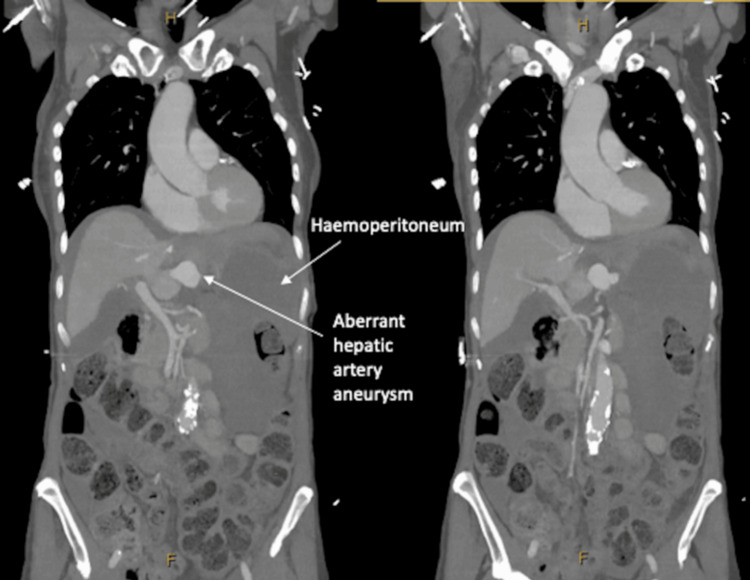
Coronal phase of CT aortogram demonstrating haemoperitoneum and aberrant left hepatic artery aneurysm.

A 71-year-old woman presented to a regional hospital with a two-day history of epigastric pain radiating to the back and nausea. HAA risk factors included hypertension and active smoking with a 20-pack-year history. Initial examination and biochemistry were unremarkable; however, six hours later, she suffered a cardiac arrest with a haemoglobin drop (Table 1). Cardiopulmonary resuscitation, blood products, and tranexamic acid were administered. Computed tomography (CT) aortogram demonstrated haemoperitoneum from a bleeding aberrant left hepatic artery (LHA) aneurysm and liver infarct (Figures 1-3).

**Table 1:** Pathology results ALT: alanine aminotransferase, AST: aspartate aminotransferase, ALP: alkaline phosphatase, GGT: gamma-glutamyl transferase

Pathology	Result	Reference interval
Haemoglobin	120-> 66-> 113	128-175g/L
Bilirubin	9	<21 micromol/L
ALT	332	5-40 units/L
AST	278	5-35 units/L
GGT	39	5-50 units/L
ALP	82	35-130 units/L

On arrival to our tertiary center, she was tachycardic and tachypneic without abdominal peritonism. Her haemoglobin improved although she developed newly deranged liver function tests (LFTs) (Table 1). Given her aberrant anatomy and instability, a decision for surgery rather than angio-embolisation was made.

Two litres of blood were evacuated upon laparotomy. The pars flaccida and left triangular ligament were divided with antero-medial liver rotation for exposure. The ruptured LHA aneurysm was 5cm in length and 2cm wide but was not actively bleeding. As per the CT, the LHA aneurysm originated from the left gastric artery (LGA), which branched off the aorta instead of the coeliac axis. It was ligated and excised with 0 vicryl ties and ligaclips, then oversewn with a running 3-0 prolene suture. Extensive washout was performed before closure.

She was closely monitored post-operatively in intensive care; she had improving hepatic dysfunction as well as coagulopathy and was discharged day 13 post admission. Outpatient follow-up with LFTs at five weeks demonstrated normal liver function.

## Discussion

To our knowledge, there are no reports describing management of a ruptured HAA in a post-arrest patient. The largest cohort of HAAs was reported at 84 patients [10]. Usually asymptomatic, they are diagnosed incidentally or on autopsy [8], unlike our patient’s presentation. Contrast-enhanced CT is the gold standard diagnostic imaging [9,10], which assisted in our patient’s HAA diagnosis and treatment planning. Treatment indications include symptomatic or pregnant patients, HAAs twice the diameter of the hepatic artery, >2cm, or >0.5cm expansion on annual surveillance [2,4]. However, others advocate for repair regardless of size due to the risk of rupture and mortality [4,7].

Decision-making requires multi-disciplinary discussion with both the surgeon and interventional radiologist [11]. Factors include HAA location, aberrant anatomy, haemodynamic status, patient age, and comorbidities [3,11]. Options include endovascular versus surgical interventions [3,11]. The former includes direct embolisation with coil or liquid agent device filling, or stenting [11]. This minimally invasive technique has limited morbidity, limited hepatic devascularisation, and shorter recovery [1,2,8].

Surgery is also indicated when embolisation is unavailable, unsuccessful, or, as in our case, not feasible. Patient factors of instability and limited reserve with co-morbidities, rather than anatomical or technical factors, precluded endovascular management. Other factors include HAA characteristics such as giant aneurysms [3,12]. Surgical options include HAA resection with bypass (saphenous vein) grafting, or HAA excision and ligation. Other procedures such as aneurysmectomy with end-to-end anastomosis, aneurysmorrhaphy, or a one-step technique with a linear vascular stapler to exclude HAA with resultant retrograde thrombosis haven't been reported [2,6].

Two cases report cholecystectomy also performed for right HAA and common HAA due to the risk of gallbladder necrosis [3,12]. To prevent hepatic ischaemia, it is also important to consider the adequacy of collaterals [3,12]. Given our patient’s aberrant left HAA and non-ischaemic liver intra-operatively, vascular reconstruction and cholecystectomy were not required.

In a recent cohort study, HAA surgical complications were higher than endovascular technique at 38% versus 20%, respectively [10]. These include pneumonia, pulmonary embolus, hepatic artery graft thrombosis, bile leak, abdominal abscess, and bowel perforation [10]. Endovascular complications include failed attempt, endoleak, haemorrhage, liver necrosis, stent dislocation, and common femoral artery pseudoaneurysm [10]. A statistically significant higher short-term morbidity in the operative group, as well as one post-operative mortality in a ruptured HAA, was identified [10].

The extensive collateral blood supply in the liver mitigates the potential risk of hepatic ischaemia. This was confirmed in our patient’s follow-up investigations, reporting normal hepatic function.

## Conclusions

This case adds to the growing body of literature, highlighting the substantial risk of HAA rupture and mortality. In patients presenting with abdominal pain, the rare diagnosis of HAA and aberrant anatomy should be considered and investigated. Prompt surgical referral for definitive management is required in a ruptured aneurysm. HAA management is variable and depends on clinical presentation, HAA characteristics, and anatomical variation. Multi-disciplinary decision-making with endovascular versus surgical treatment with or without vascular reconstruction is an ongoing challenge. Surveillance of hepatic function and new HAA development is recommended with biochemical investigations for synthetic liver function and CT scans to assess for recurrence. Future research directions include prospective comparative studies with endovascular versus surgical outcomes in HAA patients.
